# The Correlation Between Smartphone Use and Compressive Ulnar Neuropathy at the Elbow: A Retrospective Study

**DOI:** 10.3390/jcm15052004

**Published:** 2026-03-05

**Authors:** Gianmarco Vavalle, Chiara Barbieri, Davide Messina, Silvia Pietramala, Lorenzo Rocchi, Camillo Fulchignoni

**Affiliations:** 1 Department of Orthopedics and Geriatric Sciences, Catholic University of the Sacred Heart, Largo Francesco Vito, 8, 00168 Rome, Italy; davidemessina.95@libero.it (D.M.); silvia.pietramala01@icatt.it (S.P.); lorenzo.rocchi@policlinicogemelli.it (L.R.); camillo.fulchignoni2@guest.policlinicogemelli.it (C.F.); 2 Department of Orthopedics, Ageing and Rheumatological Sciences, Fondazione Policlinico Universitario A. Gemelli IRCCS, Largo Agostino Gemelli 8, 00168 Rome, Italy

**Keywords:** cubital tunnel syndrome, ulnar neuropathy, smartphone use, elbow flexion, peripheral nerve compression, digital health

## Abstract

**Background:** Cubital Tunnel Syndrome (CuTS) is the second-most common compressive neuropathy of the upper limb, traditionally associated with prolonged elbow flexion, trauma, or anatomical constraints. With the widespread adoption of smartphones, sustained upper-limb postures have emerged as potential novel risk factors for ulnar nerve compression. This retrospective study aimed to investigate the potential correlation between smartphone use patterns and the development of CuTS. **Methods:** A retrospective observational study was conducted on 100 subjects recruited between 2021 and 2024, including 50 patients with EMG-confirmed CuTS who underwent surgical decompression and 50 matched controls without clinical or electrophysiological evidence of ulnar neuropathy. Demographic variables, daily smartphone use (h/day), predominant activity type, and habitual posture during device handling were collected through clinical records and questionnaires. Group comparisons were performed using *t*-tests and Chi-square analyses, with significance set at *p* < 0.05. **Results:** Daily smartphone use was higher in the CuTS group compared with controls (4.94 ± 1.8 vs. 4.04 ± 1.5 h/day), although the difference did not reach statistical significance (*p* = 0.0716). Posture during device use showed a significant association with CuTS: 82% of affected patients reported using smartphones with the elbow flexed, compared with 56% of controls, whereas supportive postures were less frequent among CuTS patients (16% vs. 38%) (*p* = 0.019). No significant differences were found between groups regarding smartphone activity type (*p* = 0.858). **Conclusions:** Smartphone use may contribute to ulnar nerve compression primarily through ergonomically disadvantageous postures, particularly sustained elbow flexion, rather than total usage time. These findings highlight a modifiable behavioral risk factor relevant to the rising prevalence of CuTS in the digital era. Increased clinical attention to device-handling habits and public-health strategies promoting ergonomic posture may support CuTS prevention. Prospective and biomechanically informed studies are warranted to further elucidate causal mechanisms. Unmeasured confounders (e.g., occupational and sleep-related elbow flexion) may influence these associations.

## 1. Introduction

Ulnar neuropathy at the elbow, commonly known as Cubital Tunnel Syndrome (CuTS), is a frequent compressive mononeuropathy often resulting in pain, paresthesia (numbness or tingling) in the ring and 5th digit, as well as the dorsoulnar aspect of the hand, and muscle weakness in the hand. The condition arises from increased pressure on the ulnar nerve as it passes superficially through the cubital tunnel at the medial aspect of the elbow. Symptoms are frequently exacerbated at night or during specific joint positions and movements, particularly with elbow flexion. These manifestations can significantly impair quality of life and range in severity from mild weakness to marked loss of fine motor control [1]. Ulnar nerve-related pain may result from compression at multiple potential sites, including the cervical nerve roots as they exit the spinal cord, the brachial plexus, the thoracic outlet, or more distally along the upper extremity within the arm, elbow, forearm, or wrist [2].

Clinical diagnosis of Cubital Tunnel Syndrome (CuTS) is mainly based on provocative maneuvers reproducing ulnar nerve symptoms as Tinel’s sign, elbow flexion–compression test, and ulnar nerve palpation. The scratch collapse test has been described [3], but despite acceptable interrater reliability it has limited sensitivity and insufficient reliability for peripheral neuropathy diagnosis, even if specificity may be higher than other tests [4]. Ultrasound can complement clinical assessment by detecting ulnar nerve enlargement (increased cross-sectional area) around the elbow and may be useful for rapid evaluation and follow-up [5]. Overall, because no single test is universally accepted, CuTS diagnosis should combine clinical suspicion, physical examination, and confirmatory testing [5].

Management is stepwise: conservative treatment is generally used for mild-to-moderate cases (including activity modification and, when appropriate, splinting), while surgery is considered for persistent or severe symptoms and/or objective deficits. The main surgical options are in situ decompression and anterior transposition of the ulnar nerve [6].

**Figure 1:**
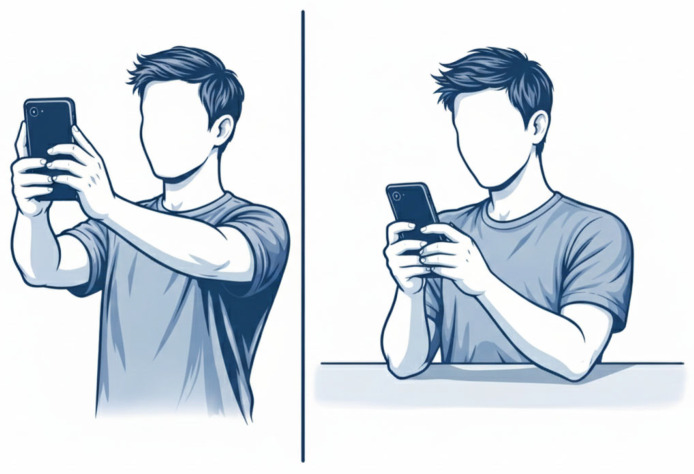
Postures during smartphone use (Image generated with ChatGPT (GPT-5.3) tool).

While classic etiologies include prolonged or repetitive elbow flexion, direct trauma, and anatomical anomalies, the dramatic rise in smartphone usage has introduced new potential risk factors. Prolonged smartphone use for activities like gaming, scrolling, or texting, can lead to various musculoskeletal disorders, including what has been colloquially termed “Text Claw” or “Cell Phone Elbow.” These non-medical terms describe symptoms such as cramping, aching, and pain in the upper extremities, including the neck, shoulders, arms, and hands, that stem from sustained, often static, and repetitive motions which can cause musculoskeletal disorders [7,8,9]. Cell phone elbow/prolonged-phone-posture (PPP) occurs due to bending or flexed posture of the elbow for long period of time, while using the phone for audio calls [10]. Recent clinical observations suggest a strong correlation between the extensive duration of cell phone use and increased pressure on the ulnar nerve [11,12,13]. Specifically, maintaining a flexed elbow posture, often near the ear for calls or held in front of the body for prolonged interaction, significantly increases mechanical tension and compression within the cubital tunnel, potentially leading to nerve compromise [14]. Despite the growing prevalence of smartphone-related upper extremity complaints, including symptoms mirroring Cubital Tunnel Syndrome, research specifically investigating this relationship remains limited compared to studies on computer-related disorders [15]. The aim of this retrospective study was to explore the association between smartphone use patterns (daily usage duration, handling posture as shown in Figure 1, and primary activity) and the development of compressive ulnar neuropathy at the elbow compared with controls. Given the multifactorial nature of CuTS, this analysis is intended to be hypothesis-generating.

## 2. Materials and Methods

The investigation was performed in full accordance with the ethical guidelines set forth in the Declaration of Helsinki.

During the preparation of this manuscript/study, the authors used ChatGPT (GPT-5.3) for the purposes of generating Figure 1. The authors have reviewed and edited the output and take full responsibility for the content of this publication.

### 2.1. Study Design

This retrospective observational study was conducted at the Orthopedic and Hand Surgery Unit of the “Fondazione Policlinico Universitario A. Gemelli IRCCS (Rome, Italy)” between January 2021 and December 2024. The primary objective was to evaluate the correlation between smartphone use and the onset of compressive ulnar neuropathy at the elbow, or Cubital Tunnel Syndrome (CuTS). The study design and reporting followed the STROBE guidelines for observational studies.

### 2.2. Population

A total of 100 subjects were included and divided equally into two groups: Group A (CuTS group): 50 patients diagnosed with Cubital Tunnel Syndrome, confirmed by electromyographic (EMG) testing, who subsequently underwent surgical decompression. Group B (Control group): 50 randomly selected subjects without any clinical or electromyographic evidence of ulnar nerve compression. Controls were chosen to be broadly comparable to the CuTS group in terms of age distribution and general demographic profile.

Inclusion criteria were: age over 18 years, right- or left-hand dominance clearly defined, and regular smartphone use for at least one year.

Exclusion criteria included: previous elbow surgery or trauma, other local musculoskeletal or neurological disorders, ulnar nerve subluxation or dislocation, systemic neuropathic conditions (e.g., diabetes), and lack of smartphone use.

### 2.3. Data Collection

Clinical records were manually and independently reviewed by three authors to ensure the accuracy and completeness of the collected data. Demographic and clinical information was retrieved from hospital records and supplemented with patient questionnaires. The following variables were recorded for each participant:

- Age, sex, and body mass index (BMI);
- Smoking and occupational status;
- Dominant limb;
- Daily duration of smartphone use (h/day);
- Type of predominant activity (calls, social media, texting, gaming);
- Usual posture during smartphone use (elbow flexed near ear, forearm resting on surface, alternating hands).

We did not collect objective or detailed measures of occupational elbow-loading intensity, sleeping posture, or other non-smartphone activities involving sustained elbow flexion (e.g., reading), which may act as confounders.

The questionnaire included both quantitative and qualitative items aimed at assessing smartphone-related ergonomic behaviors and potential risk factors. In Group A, electromyographic (EMG) findings were reviewed to confirm the diagnosis. The mean duration of daily smartphone use was self-reported and expressed as the average number of hours per day.

### 2.4. Statistical Analysis

Descriptive statistics were used to summarize the data. Continuous variables were expressed as mean ± standard deviation (SD), and categorical variables as frequencies or percentages.

Comparisons between groups were performed using the unpaired Student’s *t*-test for continuous variables and the Chi-square test for categorical variables. These analyses were performed as univariable comparisons. We acknowledge that multivariable logistic regression (e.g., including posture, h/day, age, BMI, smoking, and occupational factors) would be more appropriate to estimate independent associations and adjust for confounding; however, this study was designed as an exploratory analysis and occupational exposure was not quantified in terms of elbow-loading intensity.

A *p*-value < 0.05 was considered statistically significant. All analyses were conducted using IBM SPSS Statistics version 26.0 (IBM Corp., Armonk, NY, USA).

## 3. Results

### 3.1. Demographic Characteristics

The study included a total of 100 subjects, divided equally between patients with electromyography-confirmed Cubital Tunnel Syndrome (Group A) and randomly selected controls without clinical or EMG evidence of ulnar nerve compression (Group B). The mean age was 58.7 ± 12.4 years in the CuTS group and 54.9 ± 13.2 years in the control group.

The two cohorts were demographically comparable, with no significant differences in sex distribution, BMI, smoking status, employment, or other baseline characteristics (*p* > 0.05). These findings indicate that the control population was well-matched to the case group, reducing confounding related to measured baseline characteristics; however, unmeasured exposures (e.g., occupational elbow loading and sleeping posture) may still confound the association.

A detailed summary of demographic characteristics is reported in Table 1.

### 3.2. Smartphone Use Patterns

Mean daily smartphone use was higher in the CuTS group (4.94 ± 1.8 h/day) compared with the control group (4.04 ± 1.5 h/day). Although this difference did not reach statistical significance (*p* = 0.0716), it indicates nonetheless an increased overall smartphone use among patients with CuTS.

More pronounced differences emerged in posture-related variables. A flexed-elbow posture was more frequent in the CuTS group (82% in the “in the hand, elbow flexed” category) compared with controls (56%). Conversely, the use of a supporting surface during smartphone interaction was significantly less common in CuTS patients (16% vs. 38%). Importantly, elbow-flexion posture during smartphone use may represent a marker of cumulative mechanical stress exposure rather than a proven causal trigger of CuTS. When analyzed collectively, posture distributions demonstrated a statistically significant association with CuTS diagnosis (*p* = 0.019), posture distribution differed significantly between groups (*p* = 0.019).

In contrast, smartphone activity patterns did not differ significantly between groups. The distribution of video viewing, typing, and calling activities was similar between CuTS patients and controls, as confirmed by the non-significant global comparison (*p* = 0.858). These findings suggest that the type of activity performed is less influential than the posture adopted during smartphone use.

A comprehensive summary of smartphone-related variables including usage duration, handling posture, and activity type is provided in Table 2.

## 4. Discussion

The primary aim of this study was to investigate the potential correlation between smartphone use and compressive ulnar neuropathy at the elbow (Cubital Tunnel Syndrome, CuTS). Although CuTS is traditionally associated with prolonged elbow flexion, direct trauma, anatomical narrowing, or systemic neuropathies, the widespread adoption of smartphones in the past decade has introduced a new class of sustained postures and repetitive behaviors that may represent underrecognized risk factors. The results of our retrospective analysis indicate that while total smartphone time may play a contributory role, posture during device use showed a stronger univariable association with CuTS than usage duration in this cohort.

### 4.1. Interpretation of Main Findings

Our data revealed a higher daily smartphone use among patients with CuTS (4.94 h/day) compared with controls (4.04 h/day), although this difference did not reach statistical significance (*p* = 0.0716). While this suggests that duration of smartphone use alone may not be sufficient to precipitate ulnar nerve compression, the observed increase is clinically noteworthy, as greater exposure time inevitably increases the cumulative duration spent in potentially harmful upper-limb postures. Nevertheless, the most relevant difference between the two groups emerged from the posture-related variables, which demonstrated a clear and statistically significant association with CuTS. Specifically, 82% of CuTS patients reported using the smartphone with the elbow flexed, compared with 56% of controls, and supportive postures were significantly less common among affected individuals (16% vs. 38%). Such findings strongly indicate that ergonomic positioning, and particularly prolonged elbow flexion, was significantly associated with CuTS, outweighing the influence of overall daily exposure time.

The biomechanical rationale underlying this association is robust and well described in the literature. Elbow flexion is known to increase intraneural pressure, longitudinal traction, and deformation of the ulnar nerve within the cubital tunnel. Andrews et al. (2018) provide comprehensive anatomical and clinical evidence that flexion beyond 90° both narrows the tunnel and elevates compression forces, impairing intraneural blood flow and potentially compromising axonal transport [2]. Nakashian et al. (2020) similarly highlight the role of elbow position in modifying tunnel volume and neural excursion, reinforcing the concept that sustained flexion is one of the mechanical drivers of ulnar nerve irritation [1]. However, our study cannot determine whether the posture-related association reflects uniform ulnar nerve compression or more localized intraneural changes. Emerging high-resolution ultrasound (HRUS) evidence indicates substantial fascicular heterogeneity, suggesting that apparent nerve enlargement or global CSA-based metrics may mask clustered or selectively stressed fascicles. Therefore, mechanistic inferences should be made cautiously, and future imaging-based studies are warranted to evaluate fascicle-level alterations [16].

An additional interpretive point is the distinction between static and dynamic nerve compression. Static compression relates to sustained postures (e.g., prolonged elbow flexion) that increase intraneural pressure over time, whereas dynamic mechanisms involve movement-dependent factors such as repeated flexion–extension, friction/traction, or instability that may intermittently stress the nerve. Because smartphone habits can include both sustained flexion and repetitive transitions, our retrospective data cannot disentangle the relative contribution of static versus dynamic compression and this should be addressed in future studies using objective monitoring and dynamic imaging [17,18].

Recent experimental biomechanical work further strengthens this interpretation. Nagashima et al. (2022) quantified the strain applied to the ulnar nerve across varying degrees of elbow flexion [19]. They demonstrated that as the elbow progresses from extension to deep flexion, the ulnar nerve undergoes measurable elongation and increasing tension, even in anatomically normal specimens. This strain was not dependent on deformity but inherent to the mechanics of flexion itself, providing compelling evidence that posture alone can impose significant biomechanical stress on the nerve. Their findings align precisely with the behavioral patterns observed in our CuTS cohort, who predominantly reported postures requiring sustained flexion, such as holding the smartphone near the face or chest during one-handed use. Electrophysiological and clinical data also corroborate these observations. Halac et al. (2015) reported that habitual postures involving prolonged flexion were among the most consistently identified risk factors in symptomatic patients [20]. This reinforces the notion that posture-driven mechanical forces play a central role in the pathogenesis of CuTS and helps situate our findings within the broader context of clinical neuropathy research.

Longer smartphone use may increase the cumulative time spent in harmful positions, but the plausible contributing factor consistent with biomechanical literature, but not demonstrable as an independent predictor in this study, is the flexion-induced alteration in cubital tunnel biomechanics. This interpretation is entirely consistent with both our clinical findings and established physiological principles.

In this framework, elbow posture becomes the most clinically relevant variable. The frequent adoption of a flexed-elbow position during smartphone use, particularly during activities such as browsing, messaging, and video viewing, places the ulnar nerve in a position of repeated or prolonged mechanical stress. If compounded by individual anatomical predispositions (reduced tunnel volume, ligamentous stiffness, or age-related changes), even moderate exposure may be sufficient to trigger symptomatic compression. Such a model offers a rational explanation for the observed relationship between device-handling posture and CuTS, as well as the weaker association with total daily usage time.

Ultimately, this posture-dominant interpretation underscores the importance of considering ergonomics and biomechanics in evaluating contemporary risk factors for CuTS. As smartphone use continues to increase across all age groups, the relevance of these findings becomes increasingly significant for both clinicians and public health practitioners.

### 4.2. Comparison with Previous Literature

The results of the study conducted at our institute suggest a possible link between excessive smartphone use and cubital tunnel syndrome. Current literature has focused mainly on musculoskeletal problems of the hand and neck related to the use of mobile devices [21]. The study by Agarwal et al. analyzed the relationship between prolonged smartphone use and a number of orthopedic conditions, including smartphone arthritis, cell phone elbow, carpal tunnel syndrome, osteoarthritis of the carpometacarpal joints, and gamer’s thumb [22]. Carpal tunnel syndrome associated with excessive smartphone use has become very common nowadays. Risk factors for CTS include prolonged periods of marked flexion or extension of the wrist, frequent use of flexor muscles, and exposure to vibration, factors commonly associated with smartphone use, as reported by Athar et al. [23]. A cross-sectional study conducted at the Faculty of Medicine, Fırat University, revealed that the risk of CTS increases by 1.292 times for each additional hour of daily smartphone use [24]. Another condition to consider is De Quervain’s tenosynovitis. Another condition to consider is De Quervain’s tenosynovitis. According to Asad et al. [25], the frequency of De Quervain’s disease is significantly correlated with the number of text messages sent per day. As the number of messages increases, so does the incidence of a positive Finkelstein test. It goes from 40% in those who send fewer than 50 messages, to 64% in those who send 50–100, to 45.7% in those who send 100–200, and up to 80.9% in those who send more than 200. This inflammation is caused by repeated gripping, grasping, or twisting movements [25]. As explained above, the incidence of “cell phone elbow” is on the rise, especially among younger people. Increased elbow flexion, combined with prolonged maintenance of this position, puts strain on the nerve; the nerve itself can stretch from 4.5 to 8 mm. This reduces the space available for the nerve and increases the pressure inside the cubital tunnel according to Ukkirapandian K et al. [14]. Studies have shown that flexion maintained beyond 90° significantly increases the pressure inside the cubital tunnel, reducing the physiological gliding of the ulnar nerve [26,27]. The literature on peripheral neuropathies suggests that chronic mechanical stress can affect nerve physiology [17]. It is also known that during nighttime rest, the elbow may remain flexed for prolonged periods, contributing to ulnar compression [28]. The habit of using a smartphone in bed can therefore promote incorrect postures maintained during sleep, amplifying the neuropathic risk.

### 4.3. Epidemiological Implications

The excessive use of smartphones (especially among younger generations) and, above all, adopting incorrect posture could become a risk factor for ulnar neuropathy, contributing to an increase in cases of “cell phone elbow”. If confirmed, this could change the epidemiological profile of cubital tunnel syndrome, meaning it would no longer be limited to cases related to work or traditional posture. The excessive use of smartphones (especially among younger generations) and, above all, adopting incorrect posture could become a risk factor for ulnar neuropathy, contributing to an increase in cases of “cell phone elbow”. If confirmed, this could change the epidemiological profile of cubital tunnel syndrome, affecting not only cases related to work or traditional posture. The study conducted by Luca Padua et al. showed that prolonged phone posture (PPP) can alter the electrophysiological function of the ulnar nerve [29]. Of course, prevention is a key weapon in reducing the incidence of this condition. As regards the role of prevention, not all authors share the same opinion. Effective prevention could be achieved by subjecting the at-risk population to electrophysiological screening, as in the study conducted by Ukkirapandian K et al. [14]. A suggestion from various authors is to reduce the duration of time spent in an incorrect posture and to find more ergonomic positions that place less strain on the nervous system.

### 4.4. Strengths and Limitations

This study has several strengths and limitations. Among its strengths, it addresses a timely topic by examining the association between smartphone use and cubital tunnel syndrome. Collecting participant-reported smartphone usage provides insight into behavioral correlations with symptoms. The cross-sectional design offers a clear snapshot of the population, while the well-defined sample of 100 participants enables systematic assessment and lays a foundation for future longitudinal or interventional studies. However, the study also has limitations. The relatively small sample size may reduce statistical power and limit generalizability. Smartphone usage was self-reported, making it susceptible to recall bias and potential over- or underestimation. Moreover, the cross-sectional design precludes any evaluation of the longitudinal progression of cubital tunnel syndrome. In addition, relevant confounders were not quantitatively assessed, including occupational elbow loading (type/intensity/duration), sleeping posture, prior repetitive upper-limb activities, and non-smartphone sustained elbow flexion (e.g., reading or hobbies). These factors may bias the observed association between smartphone posture and CuTS. Additionally, our statistical approach was based on univariable comparisons (*t*-tests and chi-square) and therefore cannot estimate independent associations in a multifactorial condition such as CuTS. Multivariable logistic regression incorporating posture, h/day, age, BMI, smoking, and occupational exposure would be preferable to quantify adjusted effects and should be applied in larger prospective cohorts with more granular exposure assessment.

### 4.5. Future Directions

Future research should focus on prospective studies to clarify the temporal progression and relationship between the amount of time spent in the incorrect elbow flexion position and cubital tunnel syndrome. To do this, wearable inertial measurement units and continuous monitoring of elbow angle could be used. Advances in electroneurophysiology methods, including quantitative EMG, high-frequency ultrasound with elastography, and MR neurography, could give us a better understanding of the microstructural alterations in nerve tissue associated with repeated mechanical stress, with a view to developing predictive models capable of diagnosing the preclinical stages of compressive neuropathy. Randomised clinical trials dealing with ergonomic interventions such as the use of hands-free devices, elbow offloading strategies and behavioural modification to reduce the degree of continuous stress on the ulnar nerve.

## 5. Conclusions

This study provides evidence that smartphone use may contribute to the development of compressive ulnar neuropathy at the elbow, particularly through the adoption of sustained elbow-flexion postures during device handling. While total daily smartphone time showed only a non-significant trend toward higher exposure among patients with CuTS, posture-related variables demonstrated a clear and statistically significant association with the condition. These findings suggest that ergonomic factors, especially prolonged elbow flexion, represent the primary plausible contributing factor, consistent with biomechanical literature, of smartphone use on ulnar nerve compression, whereas time alone appears to play a secondary, cumulative role.

Given the near-universal prevalence of smartphone use, increased awareness of posture during device handling may have meaningful implications for the hypothesis for preventive strategies pending prospective confirmation of ulnar neuropathy. Clinicians should routinely assess smartphone habits when evaluating patients with ulnar nerve symptoms, while public-health initiatives might consider promoting ergonomic strategies such as reducing prolonged elbow flexion, using headphones, supporting the forearm during use, and alternating hands. These findings are hypothesis-generating and require confirmation in prospective studies with objective exposure measures.

Further prospective, biomechanically informed research, including objective measurement of elbow position during device use and integration of ultrasonographic assessment, will be essential to confirm these associations and to better define causal pathways. Nevertheless, the present findings highlight a modifiable behavioral factor that may be relevant in the growing incidence of compressive ulnar neuropathy in the modern digital era. These associations are hypothesis-generating and require confirmation using multivariable models. Future prospective studies should quantify occupational elbow loading, sleep posture, and other sustained elbow-flexion activities to better isolate the independent contribution of smartphone behaviors.

## Figures and Tables

**Table 1:** Demographics.

	CuTS Group (*n* = 50)	Control Group (*n* = 50)	*p*-Value
Total number of patients	50	50	/
Mean age of patients	58.7	54.9	0.141
(Range)	(22–80)	(20–75)	/
(Standard deviation)	(12.4)	(13.2)	/
BMI (kg/m^2^)	27.5	26.9	0.372
Smokers	26%	28%	0.811
Workers	50%	48%	0.858
Dominant side involved (%)	58%	/	/
Sex			
Male	34 (68%)	30 (60%)	0.532
Female	16 (32%)	20 (40%)	/

**Table 2:** Smartphone use, posture distribution, and activity distribution between groups.

Category	Variable	CuTS Group (*n* = 50)	Control Group (*n* = 50)	*p*-Value
Smartphone use	Mean h/day	4.94 ± 1.8	4.04 ± 1.5	0.0716
Posture Type	In the hand, elbow flexed	82%	56%	0.019
	In the hand, elbow extended	2%	6%	/
	On a support	16%	38%	/
Smartphone Activity	Video	46%	46%	0.858
	Typing	34%	30%	/
	Calling	20%	24%	/

## Data Availability

The data used in this study were obtained from Policlinico Universitario A. Gemelli IRCCS and restrictions apply to the availability of these data, which were used under license for the current study. Data are however available from the authors upon reasonable request.
